# The role of anthropometric indices in the prediction of non-alcoholic fatty liver disease in the PERSIAN Guilan Cohort study (PGCS)

**DOI:** 10.25122/jml-2018-0031

**Published:** 2018

**Authors:** Roya Mansour-Ghanaei, Fariborz Mansour-Ghanaei, Mohammadreza Naghipour, Farahnaz Joukar, Zahra Atrkar-Roushan, Mohammadjavad Tabatabaii, Najmeh Ghorani

**Affiliations:** 1. Gastrointestinal & Liver Diseases Research Center, Guilan University of Medical Sciences, Rasht, Iran; 2. Caspian Digestive Disease Research Center, Guilan University of Medical Sciences, Rasht, Iran

**Keywords:** anthropometric indices, non-alcoholic fatty liver disease, Persian Guilan Cohort study (PGCS)

## Abstract

**Introduction:** Non-alcoholic fatty liver disease (NAFLD) is an obesity-associated health problem that causes other liver diseases for the patient. Four anthropometric indices: body mass index (BMI), waist circumference (WC), waist-to-hip ratio (WHR) and waist-to-height ratio (WHtR) were analyzed as NAFLD predictors in the present study.

**Methods:** From the total number of individuals who referred to the PERSIAN Guilan Cohort study (PGCS) located in the north of Iran during the period of study, a total of 960 people were enrolled in the present study. NAFLD was diagnosed using through an abdominal ultrasound exam. Height, weight, WC, BMI, WHR and WHtR were later calculated. Chi-square, ANOVA and logistic regression analyses were used to analyze the risk factors.

**Results:** Out of the 960 individuals who were enrolled in the study, 597 (62.2%) were male and 363 (37.8%) were female (with an average age of 47.21 ± 7.29 years). There was a significant relationship between weight and NAFLD (P<0.001). There was also a significant relationship between BMI (OR= 8.41; 95% CI = 5.59–12.75), WC (OR= 2.67; 95% CI = 2.05–3.48), WHR (OR= 3.84; 95% CI = 2.26–6.52), WHtR (OR= 28.53; 95% CI = 6.94–117.31) and NAFLD (P<0.001). The results of the logistic regression analysis showed that WHtR, BMI and WC were effective predictors for the risk of NAFLD while WHtR played a more important role in the prediction of NAFLD.

**Conclusion:** Anthropometric indices, especially WHtR, as a simple screening tool, seem to be an important criterion for the detection of NAFLD.

## Introduction

NAFLD is a significant health concern and also the most common form of liver disease worldwide [1]. NAFLD is a condition defined by significant lipid accumulation (5–10%), especially triglycerides in the hepatocytes in the absence of significant chronic alcohol consumption (less than 20 g/day in men and 10 g/day in women) [2-4] that may cause other liver diseases for the patient such as non-alcoholic steatohepatitis (NASH), liver tissue fibrosis, cirrhosis, and even hepatocellular carcinoma (HCC) [2, 5]. According to previous studies, the prevalence of NAFLD ranges from 6.3-33% worldwide, with a median of 20% in the general population depending on the differences of the assessment methods [6]. Systematic review studies show that the prevalence of NAFLD is estimated to be 20-30% in the eastern countries [7]. The prevalence of NAFLD and NASH are reported to be between 2.9%-7.1% among the general Iranian population; however, in a previous study conducted in Shiraz, southern Iran, Lankarani et al. reported a prevalence rate of 21.5% among subjects, 53.26% of whom had a high BMI rate [8]. NAFLD is usually associated with diabetes, insulin resistance, increased blood lipids, increased blood pressure, age, metabolic syndrome, obesity and BMI [3, 9-11]. NAFLD is present in about 10%, 50% and 70% of subjects with prediabetes with BMI in the normal weight, overweight and obese range, respectively [12] and in about 20%, 40% and 70% of subjects with a metabolically unhealthy phenotype with BMI in the normal weight, overweight and obese range, respectively [13]. Another study showed that 22% of non-diabetic patients with NAFLD had normal BMI [14]. Although there may be a risk of NAFLD and its more severe form, NASH, in non-obese patients, most cases are found in obese or overweight people [15]. NAFLD is linked to obesity, with a reported prevalence as high as 80% in obese patients and only 16% in individuals with normal BMI who don’t have any metabolic risk factors [16]. BMI and WC are the most important factors for grading obesity [15]. The distribution of body fat, rather than the whole body fat mass, plays a critical role in fat metabolism. Visceral fat is a better predictor than subcutaneous fat for NASH and is related to the severity of NAFLD [17]. WC is considered an alternative marker for the visceral fat and the visceral fat is strongly associated with triglyceride accumulation in liver cells, liver inflammation and liver fibrosis. However, WC does have limits, e.g., the amount of the abdominal fat is estimated to be lower in short people compared to the tall ones [15, 18]. WC, WHR and WHtR have been considered as alternative indices for abdominal obesity in previous studies [19]. Since NAFLD can lead to the development of liver diseases and impose huge costs, it is very important to identify people at risk. The aim of the present research was to determine the prevalence of NAFLD and the role of anthropometric variables such as BMI, WC, WHR, WHtR for NAFLD prediction.

## Materials and methods

This is a descriptive-analytical cross-sectional study conducted on 960 individuals aged 35-60 years referring to the PERSIAN Guilan Cohort Study (PGCS), part of the Prospective Epidemiological Research Studies in Iran (The PERSIAN Cohort Study) [20]. The subjects were non randomly enrolled in the study using the sequential sampling method from April 2017 to September 2017 (3 days a week, about 15 people a day).

Exclusion criteria were patients with acute and chronic liver diseases, including hepatitis B and C; acute and chronic kidney diseases; cancers and alcohol consumption (20g/day for men and 10g/day for women); pregnancy; patients taking drugs that affect the liver (such as steroids, amiodarone and tamoxifen) and patients with hemochromatosis. The individuals were identified according to The PERSIAN Cohort Study protocol [20].

## Study measurement

Demographic information (age, gender, occupation, education, marital status, place of residence, smoking and a first-degree family history of obesity) were personally obtained from all subjects using a questionnaire after obtaining their consent letter and explaining the goals of the study.

Well-trained personnel measured subjects’ height (in cm) while wearing no shoes using a wall-mounted stadiometer (SECA 206, Hamburg, Germany) and weight (in kg) while wearing light clothing and no shoes using a scale (SECA 755, Hamburg, Germany). Body mass index (BMI, in kg/m^2^) was calculated for all individuals by dividing the weight in kilograms by the square of the height in meters. The subjects were classified in the following BMI groups: underweight (BMI<18.5 kg/m^2^), normal weight (BMI= 18.5-24.99 kg/m^2^), overweight (BMI= 25-29.9 kg/m^2^) and obese (BMI≥30 kg/m^2^) [21]. Generally, BMI<25 and BMI≥25 indicate normal body weight and obesity, respectively [22]. Abdominal obesity was considered as WC≥102 cm and WHR≥0.9 in men and WC≥88 cm and WHR≥0.85 in women (WHR = WC/HC) [19]. A WHtR of ≥0.5 (WHtR = WC/height) permits identifying obesity.. WHtR≥0.5 indicates an increased risk of obesity-related diseases in both genders in all races [19]. The WC and WHR were measured using a tape measure with a precision of 0.1 cm at the midpoint of the lower limit of the last rib and above the lower part of the iliac crest in the maximal hip circumference position, respectively. The fatty liver was diagnosed in all subjects using hepatic ultrasound (ultrasonics, sonixSP using an in-depth probe (3.5 to 5 MHz)). This was monitored and confirmed by two radiologists attending the Cohort Center after 8 to 10 hours of fasting. According to the ultrasound findings, the fatty liver was divided into three grades: — Mild (grade 1): a brief increase in the liver echogenicity; the diaphragm and intrahepatic vessels are clearly seen. — Medium (grade 2): moderate elevation of liver echogenicity; slightly impaired visualization of the intrahepatic vessels and diaphragm. — Severe (grade 3): increased liver echogenicity; decreased penetration of sound waves to the posterior side of the liver, insignificant or lack of visualization of the intrahepatic vessels and diaphragm [23].

**Statistical analysis**

Data were entered into SPSS 18 and analyzed using descriptive statistics (mean, standard deviation, frequency and percentage), and analytical statistics (chi-square, ANOVA and logistic regression and receiver-operating characteristic (ROC)). P-value <0.05 was considered to be significant.

**Ethics**

This research project was approved by the Ethics Committee of the Gastrointestinal and Liver Disease Research Center and Guilan University of Medical Sciences with the code IR.GUMS.REC.1394.499. All participants expressed their consent for participation in the research.

**Results**

Of the 960 individuals enrolled in this study, 597 (62.2%) were males. The mean age of women and men was 46.79 ± 7.16 and 47.48 ± 7.37 years, respectively and the overall mean age was 47.21 ± 7.29 years. Also, the mean age of the non-NAFLD and NAFLD individuals was 47.06 ± 7.48 and 47.41 ± 0.34 years, respectively. There was a significant relationship between smoking along with first-degree family history of obesity and NAFLD with odd ratios of OR= 1.54; 95% CI = 1.17- 2.03 and OR=1.67; 95% CI=1.29-2.17, respectively. The subjects’ characteristics with and without NAFLD are shown in Table 1.

The results of the present research showed that out of a total of 960 patients, 56.5% (n=542) and 43.5% (n=418) were non-NAFLD and NAFLD individuals, respectively. Also, 30.8% (n=296), 11.6% (n=111) and 1.1% (n=11) were reported as NAFLD grades 1, 2 and 3 respectively during the hepatic ultrasound,. Mean WC and WHR in men and women were 102.52 ± 10.61, 0.94 ± 0.05 and 94.95 ± 10.11, 0.96 ± 0.04, respectively. A total of 93.9% of women had WC≥88 while 22.1% of men had WC≥102 so there was a significant relationship between gender and WC (P<0.001). Also, a total of 99.4% women had WHR≥0.85 versus 83.9% men with WHR≥0.9 (P<0.001). A total of 87.3% of women and 68.8% of men had BMI≥25 (P<0.001) and 93% of subjects had WHtR≥0.5 (100% women vs. 88.6% men) (P<0.001). Table 2 shows the anthropometric indicators for individuals with and without NAFLD for men and women separately.

**Table 1:** The relationship between demographic characteristics and NAFLD

Variables	Total N (%)	Subjects without NAFLD* N (%)	Subjects with NAFLD N (%)	P value*
Gender				**P= 0.586**
male	597 (62.2)	333 (55.8)	264 (44.2)	
female	363 (37.8)	209 (57.6)	154 (42.4)	
Residence				**P=0.406**
City	685 (71.4)	381 (55.6)	304 (44.4)	
Village	275 (28.6)	161 (58.5)	114 (41.5)	
Job				**P=0.214**
Farmer	109(11.4)	73 (67)	36 (33)	
Housewife	310 (32.3)	172 (55.5)	138 (44.5)	
Employed	165 (17.2)	90 (54.5)	75 (45.5)	
Worker	133 (13.9)	71 (53.4)	62 (46.6)	
self-employed	243 (25.3)	136 (56)	107 (44)	
Education				**P=0.412**
Illiterate	61 (6.4)	30 (49.2)	31 (50.8)	
Elementary	207 (21.6)	114 (55.1)	93 (44.9)	
High school	545 (56.8)	319 (58.5)	226 (41.5)	
Academic	147 (15.3)	79 (53.7)	68 (46.3)	
*Age (years)*				**P=0.654**
35-44	382 (39.8)	218 (57.1)	164 (42.9)	
45-54	371 (38.6)	203 (54.7)	168 (45.3)	
55-60	207 (21.6)	121 (58.5)	86 (41.5)	
Smoking				**P=0.002**
Yes	294 (30.6)	144 (49)	150 (51)	
No	666 (69.4)	398 (59.8)	268 (40.2)	
Marital status				**P= 0.812**
Single	29 (3)	17 (58.6)	12 (14.4)	
Married	931 (97)	525 (56.4)	406 (43.6)	
A first-degree family history of obesity				**P<0.001**
Yes	427 (44.5)	211 (49.4)	216 (50.6)	
No	533 (55.5)	331 (62.1)	202 (37.9)	

* NAFLD, non-alcoholic fatty liver disease; P value<0.05

The mean weight of the NAFLD and non-NAFLD individuals was 84.23±13.18 and 71.36±10.77, respectively, with the overall weight of 76.96±13.48. There was also a significant relationship between body weight and NAFLD (P<0.001). In this study, a total of 6 (0.6%), 232 (24.2%), 434 (45.2%) and 288 (30%) subjects had BMI<18.5 (NAFLD incidence rate =0%), 18.5-24.99 (NAFLD incidence rate = 12.5%), 25-29.99 (NAFLD incidence rate = 42.6%) and BMI>30 (NAFLD incidence rate = 70.8%), respectively.

The mean BMI, WC, WHR, WHtR of the NAFLD and non-NAFLD individuals were 30.02±3.62 versus 26.02±3.53, 103.54±10.45 versus 93.40±9.10, 0.96±0.05 versus 0.93±0.04, 0.62±0.07 versus 0.56±0.07, respectively. The findings of the present study showed that the risk of NAFLD incidence is eight times higher in individuals with BMI ≥25 compared to those with BMI< 25 and individuals with higher WC, WHR, WHtR and weight were at higher risk of NAFLD (Table 3). The results of the current research study also showed that WHtR (OR= 5.74), BMI (OR= 5.67) and WC (OR= 1.53) are three effective indices for the prediction of NAFLD (Table 4). The ROC curve displays the performance of anthropometric indicators in predicting NAFLD (Diagram 1). The present study revealed that with an increase in the mean BMI, WC, WHR, WHtR, the degree of fatty liver was increased. Diagram 2 shows the relationship between different grades of fatty liver with BMI, WC, WHR, WHtR.

**Table 2:** Anthropometric indicators for individuals with and without NAFLD for men and women separately

Variables	Female		P value* OR(CI)	Male		P value* OR(CI)
	without NAFLD*	with NAFLD		without NAFLD	with NAFLD	
BMI*			P<0.001			P<0.001
<25	41(83.7)	8(16.3)	ref	168(88.9)	21(11.1)	ref
≥25	168(53.5)	145(46.5)	4.45(2.02-9.80)	165(40.4)	243(59.6)	11.78 (7.18–19.32)
WC*, cm			P<0.001			P<0.001
<102 for Male/88 for Female	20(90.9)	2(9.1)	ref	308(67.4)	149(32.6)	ref
≥102 for Male/88 for Female	189(55.4)	152(44.6)	8.04 (1.85-34.94)	22(16.9)	108(83.1)	10.14(6.16–16.70)
WHR*			P=0.51			P<0.001
<0.9for Male/0.85 for Female	2(100)	0(0)	ref	78(81.3)	18(18.8)	ref
≥0.9 for Male/0.85 for Female	207(57.3)	154(42.7)	1.74(1.59-1.90)	255(50.9)	246(49.1)	4.18(2.43–7.18)
WHtR*			P-			P<0.001
<0.5	0(0)	0(0)	ref	64(97)	2(3)	ref
≥0.5	207(57.3)	154(42.7)	-	255(49.7)	258(50.3)	32.37(7.84–133.67)

* P value<0.05; NAFLD, non-alcoholic fatty liver disease; Body mass index (BMI); Waist circumference (WC); Waist-to-hip ratio (WHR); Waist-to-height ratio (WHtR)

**Table 3:** Relationship between anthropometric indicators and NAFLD

Variables	Total N (%)	Subjects without NAFLD N (%)	Subjects with NAFLD N (%)	P value*	OR(CI)
*BMI				
<25	238 (24.8)	209 (87.8)	29 (12.2)	P<0.001	(ref)
≧25	722 (75.2)	333 (46.1)	389 (53.9)		8.41 (5.59-12.75)
*WC, cm				
<102 for Male/88 for Female	479 (50.43)	328 (68.5)	151 (31.5)	P<0.001	(ref)
≧102 for Male/88 for Female	471 (49.57)	211 (44.8)	260 (55.2)		2.67 (2.05-3.48)
*WHR				
<0.9for Male/0.85 for Female	98 (10.21)	18 (18.4)	80 (81.6)	P<0.001	(ref)
≧0.9 for Male/0.85 for Female	862 (89.79)	400 (46.4)	462 (53.6)		3.84 (2.26-6.52)
*WHtR				
<0.5	66 (7)	64 (97)	2 (3)	P<0.001	(ref)
≥0.5	874 (93)	462 (52.9)	412 (47.1)		28.53 (6.94-117.31)

* P value < 0.05; NAFLD,non-alcoholic fatty liver disease; *Body mass index (BMI); Waist circumference (WC); Waist-to-hip ratio (WHR); Waist-to-height ratio (WHtR)

## Discussion

The present study showed that 43.5% of subjects suffered from NAFLD. NAFLD had a prevalence rate of 10-30% globally [24] and 46% in the United States[25]. In a previous systematic review study conducted in Asia, the prevalence of NAFLD was estimated to be 15-20% [26]. The prevalence of NAFLD was reported to be 42% [27], 15.3% [28], 43.8% [29], 37.2% [30] and 21.5% [8] in studies carried out in Hong Kong, southern Iran, northern Iran and in Lankarani *et al*.’s study, respectively. The results of the present study revealed that first-degree family history of smoking and obesity was correlated with NAFLD, but there was no relationship between other demographic characteristics such as age, gender, marital status, occupation, education and NAFLD. The results of another study conducted in Sudan also showed no gender differences in the development of NAFLD [31]. The results of an Indian study reported a NAFLD incidence rate of 8.7% among 1911 subjects and no relationship was found between age, gender or job and NAFLD [32]. Also, the results of a study in southern Iran showed no relationship between education, occupation and smoking and NAFLD [33]. However, some previous studies showed that there was a relationship between gender [29, 33-35], age [31, 33, 34] and marital status [33] and NAFLD. In the current study, NAFLD was seen in 12.5% of individuals with normal BMI. The results of a study on 162 NAFLD patients conducted in Greece showed that NAFLD was prevalent among 12% of those with normal BMI [36]. Another study in South Korea also reported that 12.5% of non-obese subjects had NAFLD [22]. The results of a study on 205 NAFLD and 131 non-NAFLD individuals in India showed that 13.2% of the NAFLD patients were underweight, 68.8% obese and 18% overweight [37]. The results of a study in Iran showed that out of 95 (19.9%) of the individuals diagnosed with NAFLD, 2.7% (n=13) were underweight and 17.2% (n=82) had BMI≥25 [33]. This research showed a significant relationship between anthropometric indices (BMI, WC, WHR, WHtR) and NAFLD. Hence, WHtR, BMI and WC are three important indices for the prediction of NAFLD. The incidence of NAFLD in obese subjects and those with NASH and cirrhosis was more than 60-90%, 20-25% and 8-2%, respectively [38]. The NAFLD prevalence seems to be strongly correlated with overweight and central obesity (abdominal obesity) [39]. The body fat distribution is more important than that of the whole body fat mass, and visceral fat is a better predictor of steatohepatitis compared to the subcutaneous fat and this is associated with the severity of NAFLD while WC is an indicator of visceral fat and is associated with fat accumulation in liver cells, inflammation and liver fibrosis [15, 17]. The results of a study on 916 patients with NAFLD in China showed that there was a strong positive relationship between BMI (and not WC) and NAFLD. Patients with higher BMI and WC are more at risk of NAFLD [15]. The results of another study on 250 NAFLD and 240 non-NAFLD individuals in China reported that WHR was a more effective factor, instead of WC, for the prediction of NAFLD and, that BMI, compared to WC, was also reported to have a higher impact on NAFLD prediction. In addition, central obesity had a positive effect on the incidence of NAFLD in obese subjects [40]. Many studies have shown that there is a strong correlation between BMI and NAFLD and that people with higher BMI are at greater risk of NAFLD. They also referred to weight loss as an important strategy for preventing and managing NAFLD [31, 33, 41, 42]. Furthermore, in a community-based longitudinal cohort study on 6403 participants, Miyake *et al*. showed that BMI was the most important predictor for the risk of NAFLD onset in both genders [43]. The results of a study in India showed that people with BMI>25 (OR=4.3, 95% CI = 1.6-11.5) and abdominal obesity (OR = 3.6, 95% CI = 1.7-7.2) were at higher risk of NAFLD and that central obesity was considered as a risk factor for NAFLD in underweight subjects [32]. Fung *et al*. also referred to WC as the most important factor associated with NAFLD [27]. In his study, Ostovaneh showed that 38.2% of patients with NAFLD and normal BMI had high abdominal obesity and WHR. Central obesity indices such as WC and WHR are correlated with the visceral obesity [44] and there is also a strong relationship between the central obesity indices and NAFLD, cardiovascular disorders and other obesity-related complications. It is suggested that obesity is assessed not only by using BMI but also central obesity indicators such as WC and WHR [45]. WHR is the most important anthropometric index for NAFLD and has been considered as an independent factor risk in previous studies [40, 46]. Height is an important parameter that should be taken into consideration before obesity is taken into account, since it may affect the accumulation of obesity or its distribution [47]. Taller people, unlike obese people, might have a lower risk for cardiometabolic diseases [48]. Many studies revealed that there is a strong relationship between NAFLD and WHtR, and referred to WHtR as a useful parameter in predicting NAFLD [47, 49, 50].

**Table 4:** Predictors of NAFLD in the logistic regression model

	B	S.E.	Wald	p	OR	95%CI
**WHTR**	1.749	0.788	4.932	0.026	5.748	1.22-26.90
**BMI**	1.737	0.244	50.850	<0.001	5.678	3.52-9.15
**WC**	0.425	0.152	7.800	0.005	1.530	1.13-2.06
**WHR**	-0.090	0.353	0.064	0.800	0.914	0.45-1.82
**CONSTANT**	-3.506	0.725	23.410	<0.001	0.030

## Conclusion

Since NAFLD can lead to the development of severe liver disease, it is vital to identify those at risk of the disease and anthropometric indices seem to be very important as a criterion.

**Figure 1:**
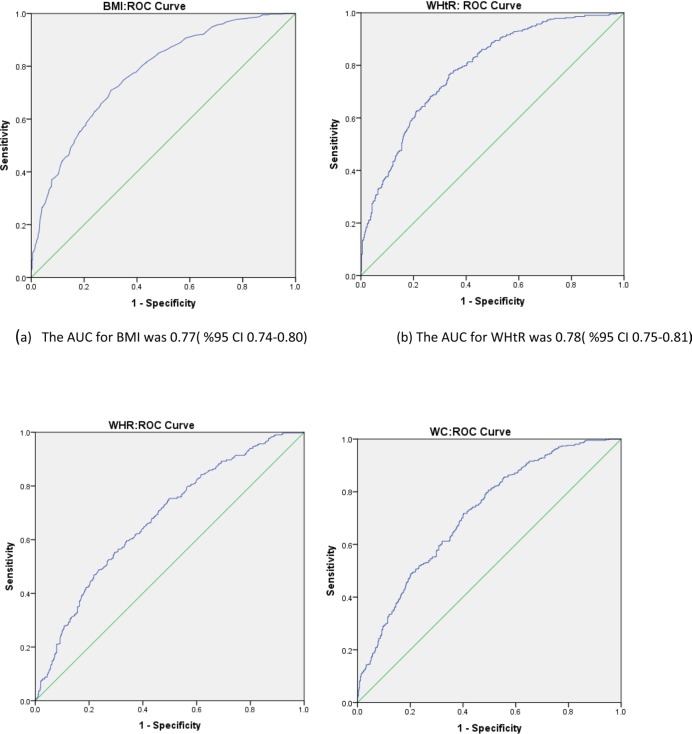
The ROC curve displays anthropometric indicators for NAFLD prediction. (a) Body Mass Index (BMI) (b) Waist-to-height ratio (WHtR) (c) Waist-to-hip ratio (WHR) (d) Waist circumference (WC)

According to the present study, WHtR was determined to be the most important predictor of NAFLD risk and a simple screening tool for NAFLD detection.

One of the limitations of this study is the use of ultrasound (a simple and low-cost method) to identify NAFLD instead of liver biopsy. Liver biopsy is the golden standard but an invasive method for NAFLD diagnosis. On the other hand, there is a risk of possible overlapping of near-grades of NAFLD in the ultrasound method, because the visual rating is dependent on the operator. To resolve this limitation, the views of two radiologists were simultaneously used to identify NAFLD.

## Acknowledgments

This study is a part of Ph.D. research thesis at the Gastrointestinal & Liver Diseases Research Center (GLDRC). We would like to thank all members of the Gastrointestinal & Liver Diseases Research Center (GLDRC) and the PERSIAN Guilan Cohort Study (PGCS).

**Figure 2:**
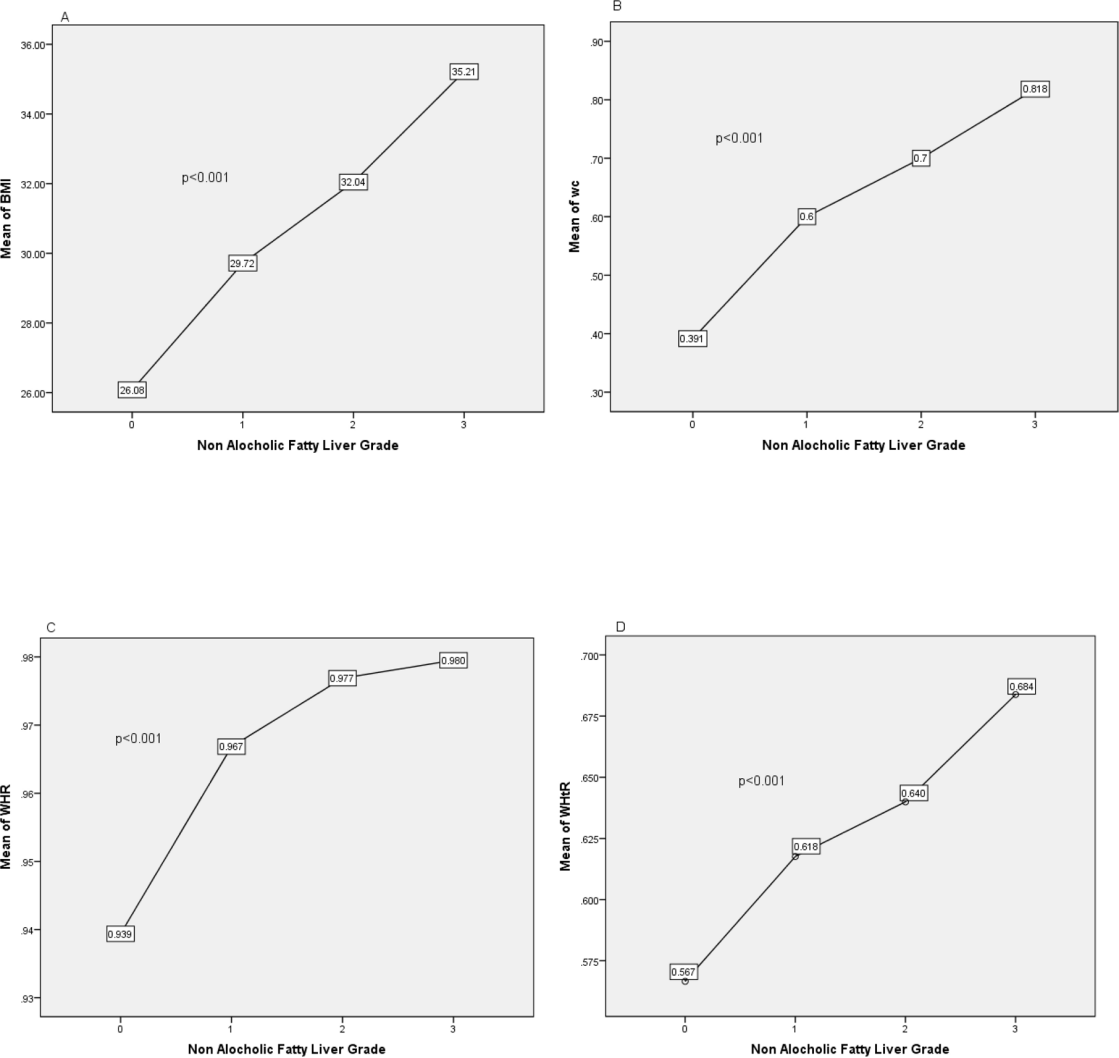
Average anthropometric characteristics versus NAFLD grades (a) Body Mass Index (BMI) versus NAFLD Grades (b) Waist circumference (WC) versus NAFLD Grades (c) Waist-to-hip ratio (WHR) versus NAFLD Grades (d) Waist-to-height ratio (WHtR) versus. NAFLD Grades

## Conflict of Interest

The authors confirm that there are no conflicts of interest.
